# Extensive skin necrosis following total hip arthroplasty performed through the direct anterior approach

**DOI:** 10.1186/s12891-023-06643-z

**Published:** 2023-06-23

**Authors:** Nimatullah Idris, Matthieu Zingg, Morgan Gauthier, Carlo M. Oranges, Daniel F. Kalbermatten, Didier Hannouche

**Affiliations:** 1 Department of Orthopaedic Surgery, Geneva University Hospitals & Faculty of Medicine, Avenue Gabrielle Perret Gentil 4, Geneva, 1205 Switzerland; 2 Department of Plastic, Reconstructive and Aesthetic Surgery, Geneva University Hospitals & Faculty of Medicine, Geneva, Switzerland

**Keywords:** Total hip arthroplasty (THA), Skin necrosis, Wound complication, Periarticular infiltration (PAI), Anterolateral thigh flap

## Abstract

**Background:**

Total hip arthroplasty is a widely performed surgical procedure, which enables patients to regain mobility, alleviates pain, and improves overall quality of life. Periarticular multimodal drug infiltration (PAI) is increasingly being used as an effective postoperative pain management, decreasing the systemic consumption of opioids. Extensive postoperative skin necrosis without a deep joint infection as a complication of total hip arthroplasty with PAI has not yet been described.

**Case presentation:**

A 71-year-old patient who underwent total hip arthroplasty of the right hip for primary osteoarthritis through the Direct Anterior Approach presented postoperatively a large area of necrotic skin at the incision. Joint infection was excluded. An extensive debridement was performed and the tissue defect was reconstructed by a pedicled anterolateral thigh flap. The skin maintained a satisfactory appearance at 1 year postoperatively, and the hip was pain-free with restored ranges of motion. The patient was able to walk with no support and without limitation.

**Conclusion:**

We address the possible risk factors, discuss the use of epinephrine in PAI and explore possible treatment options for such a complication.

**Supplementary Information:**

The online version contains supplementary material available at 10.1186/s12891-023-06643-z.

## Background

Total hip arthroplasty (THA) is a common surgical treatment which provides reliable functional outcomes in patients suffering from hip osteoarthritis. Effective postoperative pain control is most important to enable prompt rehabilitation, reduce postoperative complications, and reduce the length of hospital stay. Pain control is achieved by several methods, including systemic treatments with Non-Steroidal Anti-inflammatory Drugs, opioids, and regional treatments such as epidural anaesthesia, peripheral nerve blocks, and periarticular multimodal drug infiltration (PAI). Up until recently, the efficacy of PAI in pain relief following total hip arthroplasty during rest and activity, and its impact on the length of hospital stay and total opioid consumption was not fully established. A recent meta-analysis concluded that PAI showed better pain relief and less opioid consumption and is safe and effective following total hip arthroplasty [1]. However, the length of hospital stay was not different. While some studies have reported that the inclusion of epinephrine in PAI prolongs the duration of local anaesthetics, other studies have reported no significant increase in the duration of the effect when added to ropivacaine [2]. Wound complications following PAI with or without epinephrine were rarely reported and very little has been published regarding this complication. In the meta-analysis done by Ma HH et al., only two studies stated wound complications following PAI after hip arthroplasty requiring surgical debridement without further elucidation [1]. We present a case of extensive skin necrosis as a complication of THA performed through the Direct Anterior Approach with PAI. There was no concomitant deep tissue and joint infection. The patient was successfully treated with surgical debridement, antibiotics and a soft tissue reconstruction with a pedicled anterolateral thigh flap (ALT). To our knowledge, there is no publication reporting such a complication after THA.

This case is presented in accordance with the 2013 CARE checklist.

## Case Presentation

**Fig. 1:**
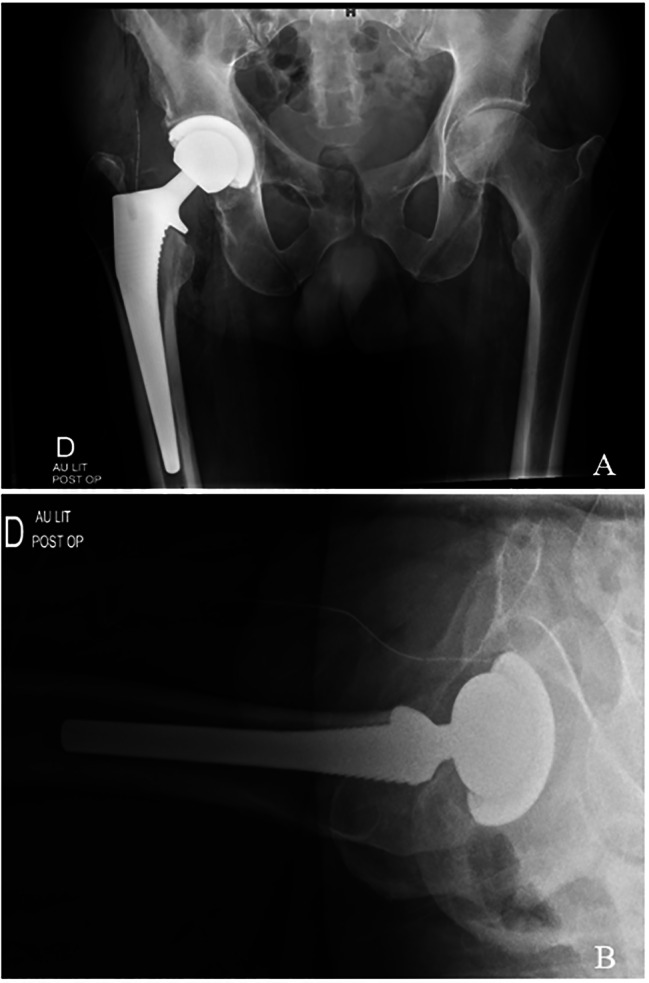
Postoperative X-Ray with Anteroposterior view (**A**) and Lateral view (**B**)

**Fig. 2:**
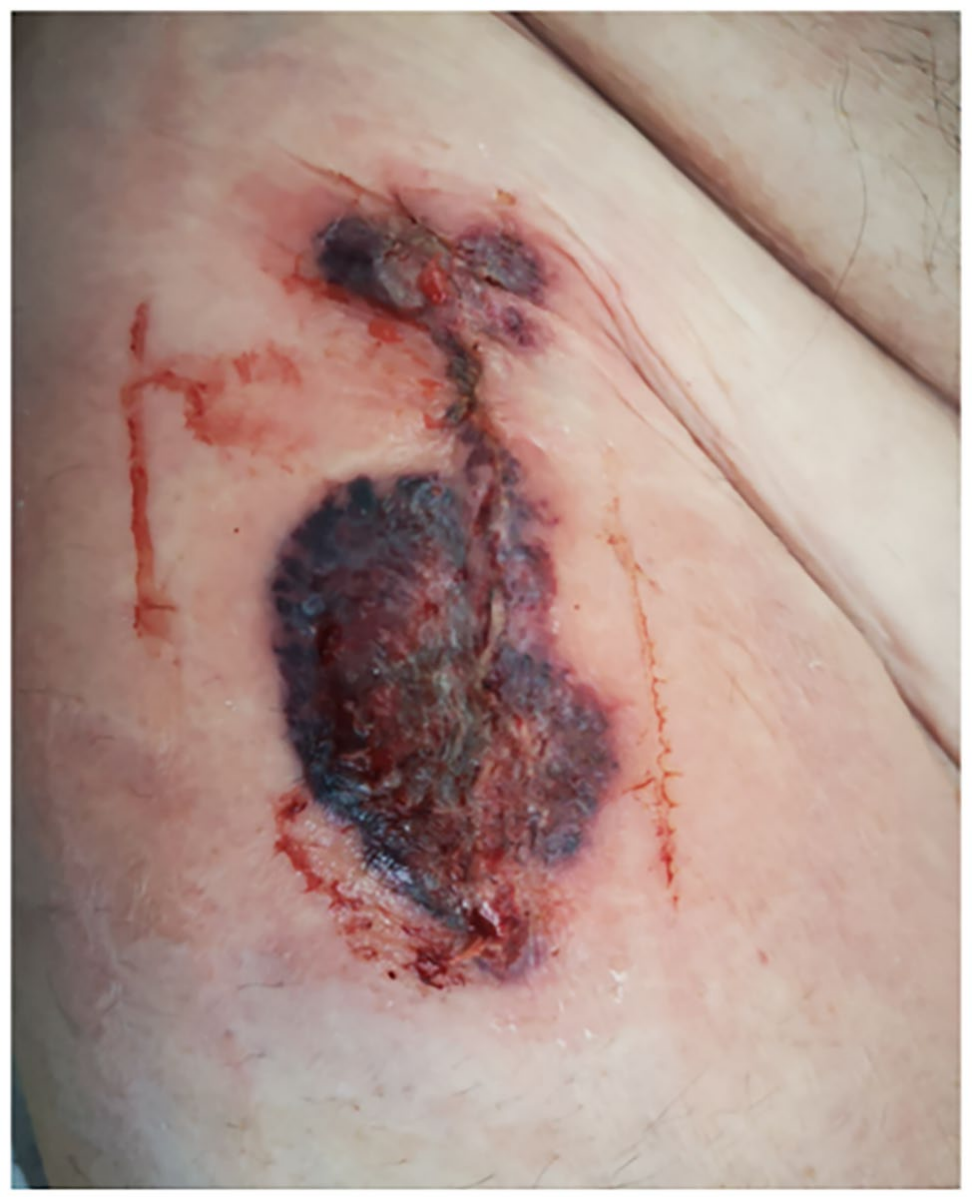
Extensive skin necrosis around the surgical wound

A 71-year-old male, non-smoker, with a previous history of cardiac ischemia was admitted for a THA. The patient was positioned in the supine position on a traction table. Surgery was performed under general anaesthesia through the Direct Anterior Approach. The patient received a fully hydroxyapatite-coated uncemented stem (Corail®; Depuy Orthopaedics Inc, Warsaw, USA), an uncemented cup (Pinnacle®; Depuy Orthopaedics Inc, Warsaw, USA), and a ceramic-highly-crosslinked polyethylene bearing surface (Fig. 1). The operation was uneventful and within usual time limits (55mn). At the end of the operation, before skin closure, 40 ml of a multimodal drug cocktail solution containing 60 mg of 0.5% levobupivacain (Chirocaine®, Abott, Rungis Cedex, France), 30 mg of ketorolac (30 mg/ml; Toradol®, Roche), and 0.1 mg of epinephrine (0,1 mg/ml; Adrenalin®, NM Pharma) was injected for perioperative pain control. The mixture was infiltrated into soft tissues as follows: 5 mL anteriorly to the rectus femoris muscle, 5 mL to the anterior capsule, 5 mL to tensor fascia lata, 5 mL to the postero-superior capsule, 10 mL to the anterior subcutaneous border, and 10 mL to the posterior subcutaneous border. Low molecular weight heparin was used to prevent deep venous thrombosis (enoxaparin SC 40 mg daily). On day 5 postoperatively, the patient presented with fever and abdominal pain and was diagnosed with acute diverticulitis. He was treated initially with an antibiotic regimen of intravenous ceftriaxone 2 g 1x/day and metronidazole 500 mg 3x/day for two days. Ceftriaxone treatment was stopped and he was started on ciprofloxacin PO 500 mg 2x/day and metronidazole was continued PO at the same doses for an additional 6 days. On day 8 postoperatively, while under the latter treatment for acute diverticulitis, the patient developed an extensive humid necrosis of the skin incision, about 6 × 9 cm in diameter, which was associated with a very mild subcutaneous hematoma (Fig. 2). The patient was apyretic. There was a local erythema surrounding the incision site without any purulent discharge. There was no acute increase in his inflammatory parameters with a white blood cell count (WBC) at 20G/l and C-reactive protein at 60 mg/l. An aspiration of the hip joint with synovial fluid analysis was conducted. The bacterial culture of synovial and blood were negative and the WBC count within the joint fluid was 4650 cells/µl. A broad range bacterial PCR analysis was also negative. Based on these evaluations, the possibility of a joint infection was excluded. An abdominal computed tomography (CT) angiography showed patent iliac and femoral vessels without significant stenosis.

**Fig. 3:**
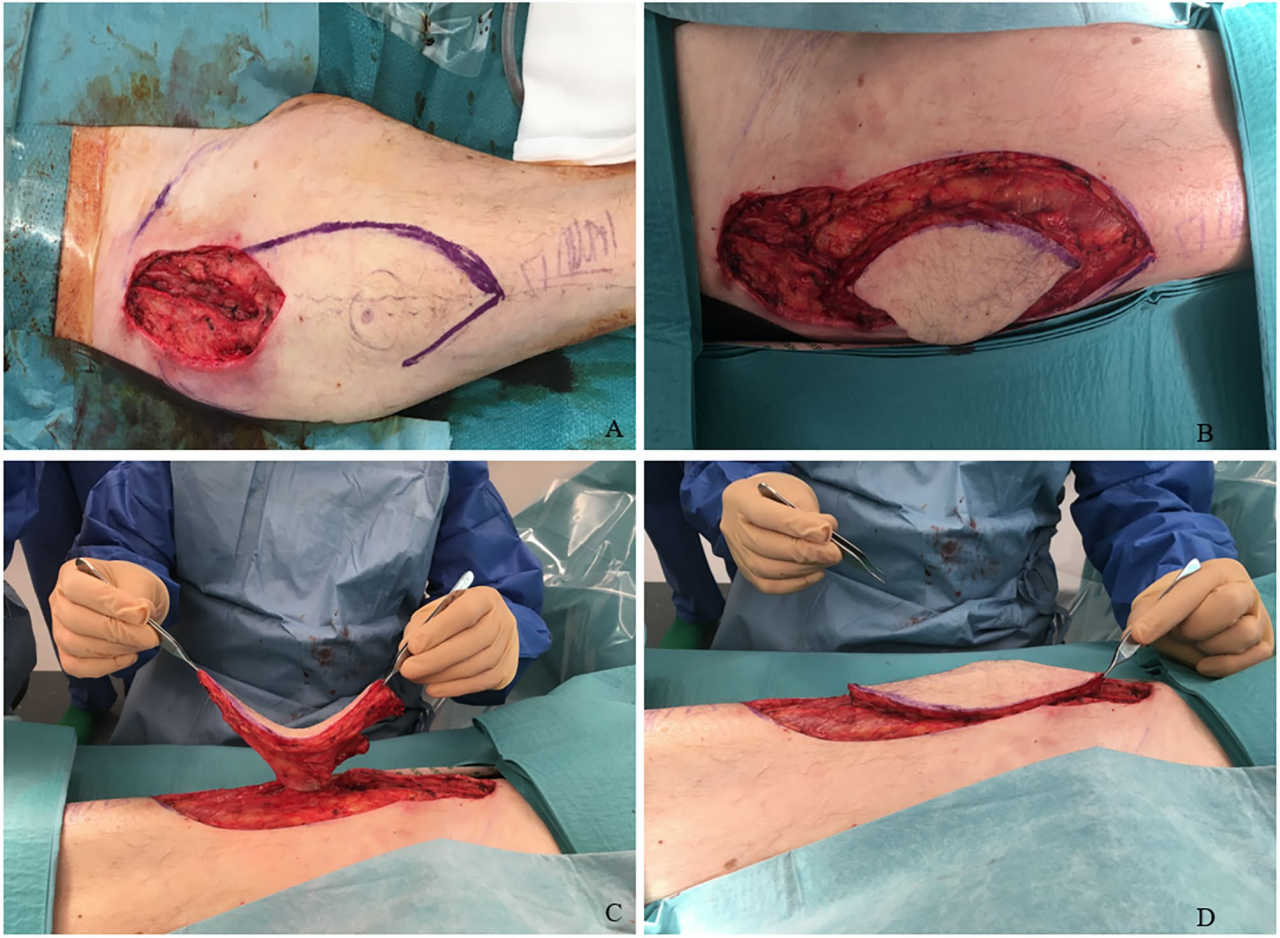
Surgical debridement and reconstruction with a pedicled ALT. Defect (**A**), ALT harvest (**B**), Pedicled ALT (**C**), Mobilization of the ALT proximally (**D**)

**Fig. 4:**
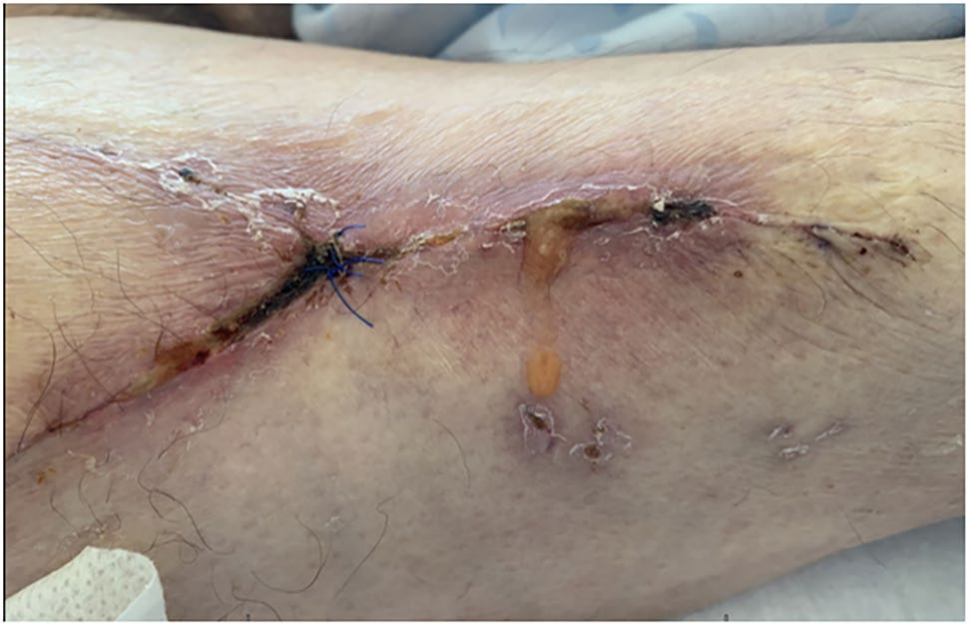
Wound dehiscence 14 days after ALT reconstruction

**Fig. 5:**
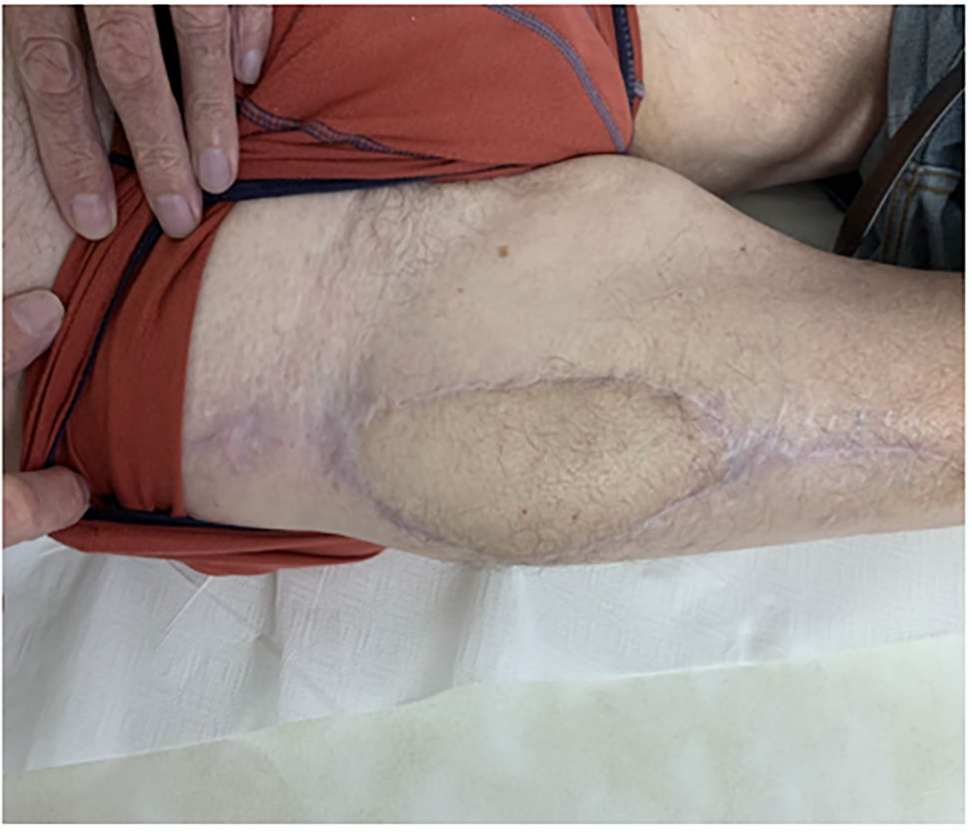
Final aspect at one year Video: Intraoperative view ALT

Considering the presence of a large skin necrosis and the high risk of infection of the underlying hip joint, we decided to perform a single-staged surgical debridement and reconstruction of the skin and soft tissue defect using a pedicled fasciocutaneous anterolateral thigh flap (ALT) of about 15 × 6 cm (Fig. 3; video). Surgical reconstruction was performed 12 days after the index procedure. The donor site was closed primarily and a suction drainage was placed. A doppler ultrasonographic control of the flap was regularly performed during the first two days and confirmed it was well vascularised. Despite initial favourable evolution, the patient presented a wound dehiscence two weeks later at the donor site without any infection and he underwent a revision surgery during which the ALT flap was mobilised and an additional skin flap was used for a tension free closure (Fig. 4). He received broad spectrum prophylactic intravenous antibiotic therapy with Piperacilline-Tazobactam 4.5 g 3x/day and Vancomycine 1 g 2x/day for twelve days. Thereafter, the surgical incisions healed well with no further complications and he began walking with support 1 week after. The patient was transferred to a rehabilitation hospital unit. The skin maintained a satisfactory appearance at 2 months and 1 year (Fig. 5). One year after the operation, hip range of motion was 90-0-0 (flexion/extension), 30-0-20 (external/internal rotation), 30-0-10 (abduction/adduction) and the patient was walking with no support. X-ray of the pelvis and hip joint showed a normal prosthesis without any signs of loosening, bone destruction or displaced prosthetic parts.

## Discussion and conclusion

Wound complications after joint replacement can result in significant morbidity to the patient with increased risk on infections, further surgeries, increased recovery period and hospital stay [3]. Wound complications following THA are less common as compared to total knee arthroplasty (TKA) [4]. Cases of wound necrosis following THA have been briefly mentioned but never been described in detail [5] and a case of skin necrosis resulting in death following THA was linked to warfarin use [6]. Other risk factors identified with wound complications such as wound dehiscence and infections following THA performed through the DAA include female sex and obese patients [3], both of which do not apply to our patient. Once a wound complication arises, differential diagnosis should be established based on patient history, clinical examination and laboratory and imaging results. Prompt medical treatment is required.

Interestingly, in our patient, the skin necrosis was first noted on day 8 postoperatively. The dressings had been changed at day 3 and day 6 and a clean wound with no oozing or redness was described. In other reported cases of skin necrosis following local anaesthetics infiltration with epinephrine, skin changes only appeared around day 5. This difference could be due to the site of injection, which was the penoscrotal junction in one case [7], and the breast periareolar skin in one case [8].

Based on expertise from our department of infectious diseases, we excluded the possibility of acute periprosthetic joint infection as the bacterial culture of synovial fluid was negative with a WBC count of 4650 cells/µl (< 10 000 cells/µl) in the joint fluid [9]. A broad range bacterial PCR analysis was also negative. The patient did not present with symptoms associated with necrotising infections [10]. During this time, the patient was under antibiotic treatment of ciprofloxacine and metronidazole for diverticulitis. However, these antibiotics are generally ineffective against bacteria commonly found in complicated skin and soft tissue infections, such as S.aureus and S. pyogens [10].

Malnutrition compromises wound healing as decreased protein reserves affects collagen synthesis and fibroblast proliferation and decreased albumin levels promotes tissue oedema formation [11]. It has been linked with surgical site and periprosthetic joint infections, wound dehiscence, and persistent wound drainage, following TKA and THA [11]. Post operatively, this patient suffered from protein energy malnutrition, with an albumin level of 29 g/l and total lymphocyte count of 1.2 G/l. He had lost 4.5 kg within the first 3 weeks of hospitalisation in a context of severe lack of appetite. It is likely that he was already malnourished prior to the THA as he already suffered from dysphagia to solids due to Zenker diverticulum.

PAI is a multimodal drug solution comprised of three active agents, long-acting local anaesthetics such as ropivacaine and bupivacaine, nonsteroidal anti-inflammatory drugs (NSAIDs) and epinephrine. Local anaesthetics block voltage-gated sodium channels in nociceptors, thereby decreasing pain transmission. Injection of NSAIDs such as ketorolac reduces pain by inhibiting the production of proinflammatory mediators such as prostaglandins. Epinephrine is a nonspecific alpha- and beta-adrenergic agonist, widely used in local anaesthesia with the rationale that through local vasoconstriction it decreases absorption thereby increasing the duration of analgesia. While some studies have reported that the inclusion of epinephrine in PAI prolongs the duration of local anaesthetics, other studies have reported no significant increase in the duration when epinephrine is added to ropivacaine due to the intrinsic vasoconstrictive effect of ropivacaine [2]. A recent prospective randomized double-blind study reported no cases of skin necrosis and wound dehiscence after TKA but reported skin colour changes in 10% of patients who received epinephrine versus 1% in the group without [2]. The role of epinephrine in skin necrosis following local anaesthesia is still controversal, with some studies supporting its role in the development of skin necrosis [7] [12]. In one of these studies, a reversal of necrosis using an alpha-receptor blocker such as phentolamine has been demonstrated [12]. According to pharmaceutical guidelines, epinephrine is contraindicated in areas with terminal vessels such as in digital and penile blocks. However, a review of literature recently published, established no evidence for a causal relationship between the use of epinephrine and necrosis of finger or penis [13].

Other possible causes for wound complications include the use of retractors and their pressure effects on skin and soft tissues. However, we have routinely performed total hip arthroplasty through the Direct Anterior Approach (DAA) for more than 12 years (approx. 400 hips/year), and we have not observed this issue previously [14]. Also, given the important volume of hip arthroplasty performed through this approach in the world, one would expect that this complication would have been reported more often if it was related to the use of retractors.

The DAA has dramatically gained in popularity over the last 15 years because it is a muscle sparing.

approach, with a potential reduction of postoperative pain and length of stay. As the skin is thinner in the anterior aspect of the hip, the risks for wound complications might be increased. However, there have been several studies, reviews and meta-analyses which compared early outcomes of total hip replacement through the main different surgical approaches, and reported similar rates of wound complications [15] [16]. Wilson et al. reported a 2.7% rate of wound dehiscence in a series of 3,687 patients who underwent a primary DAA total hip replacement between 2010 and 2019 [17]. Dehiscence, which was noted at a median of 27 days post-surgery, was more frequent in women and in obese patients [17].

In this case, we performed surgical debridement and reconstruction of the skin and soft tissue defect using a pedicled fasciocutaneous anterolateral thigh flap. This method is a reliable and versatile option for loco-regional reconstruction due to the large skin and soft tissue availability, reliable blood supply, long vascular pedicle and wide arc of rotation [18]. Furthermore, no microvascular anastomosis is required. To evaluate patency of peripheral arteries prior to the reconstruction surgery, an abdominal CT angiography was performed which showed no significant stenosis proximally and patent iliac and femoral vessels. Despite the fact that the patient developed a wound dehiscence at the donor site, wound healing was achieved within a few weeks with a good aesthetic result at 1 year. A negative pressure wound therapy as an adjuvant therapy to reconstructive surgery could have been an alternative treatment option. This method promotes tissue growth by reducing tissue oedema, inducing wound contraction, enhancing blood supply and reducing bacterial colonies [19]. It has been shown to be effective as an adjuvant therapy for defects as large as 20 × 30 cm [19] and in reducing morbidity such as wound dehiscence and seroma, following primary closure of donor-site after harvest of an ALT [20]. The use of other fasciocutaneous flaps, such as the deep inferior epigastric perforator (DIEP) flap and the Limberg transposition flap, have been described as successful alternatives for hip reconstruction [21][22]. Muscle flaps have also been largely employed, especially in case of hip prosthetic joint infection. This includes the following flaps: Vastus lateralis, Rectus femoris, Rectus abdominis, Gluteus maximus, Latissimus dorsi, and Tensor fascia lata [23].

To the best of our knowledge, this case represents the first evidence of a non-infectious and non-warfarin related extensive skin necrosis as a complication following total hip arthroplasty. In such cases, further testing should be performed to rule out possible causes such as infection and malnutrition. We achieved a good outcome with extensive debridement and wound reconstruction using a pedicled fasciocutaenous anterolateral thigh flap. As the exact cause of skin necrosis cannot be determined from a single case report, larger studies are required. Additionally, further clinical trials are needed to establish the risk and benefits of using epinephrine in PAI. Meanwhile, we decided to change our practice and not to use epinephrine anymore.

## Electronic supplementary material

Below is the link to the electronic supplementary material.

Supplementary Material 1

Supplementary Material 2

## Data Availability

The datasets used and/or analyzed during the current study are available from the corresponding author on reasonable request.

## Abbreviations

THA: Total Hip Arthroplasty
ALT: Anterolateral thigh flap
PAI: Periarticular injections
TKA: Total knee arthroplasty
