# Supplementary material for: Extensive skin necrosis following total hip arthroplasty performed through the direct anterior approach
Source: BMC Musculoskelet Disord. 2023 Jun 23;24:516. doi: 10.1186/s12891-023-06643-z (PMC10288717; doi:10.1186/s12891-023-06643-z)
Supplement: Supplementary file 1 — Supplementary Material 1 [file 12891_2023_6643_MOESM1_ESM.pdf]

CARE case report guidelines

CARE Checklist of information to include when writing a case report

(cc) BY-NC-ND

Topic	Item	Checklist item description	Reported on Line
Title	1	The diagnosis or intervention of primary focus followed by the words "case report"	Title page
Key Words	2	2 to 5 key words that identify diagnoses or interventions in this case report, including "case report"	Page 2 Lines 23-27
Abstract (no references)	3a	Introduction: What is unique about this case and what does it add to the scientific literature?	Page 2 Abstract
	3b	Main symptoms and/or important clinical findings	Page 2 Abstract, paragraph 2
	3c	The main diagnoses, therapeutic interventions, and outcomes	Page 2 Abstract, paragraph 2
	3d	Conclusion—What is the main "take-away" lesson(s) from this case?	Page 2 Abstract, lines 16-18
Introduction	4	One or two paragraphs summarizing why this case is unique (**may include references**)	Page 3 Introduction, paragraph 1
Patient Information	5a	De-identified patient specific information.	Page 3 line 29
	5b	Primary concerns and symptoms of the patient.	Page 4 paragraph 1 and 2
	5c	Medical, family, and psycho-social history including relevant genetic information	Page 3 line 29
	5d	Relevant past interventions with outcomes	NA
Clinical Findings	6	Describe significant physical examination (PE) and important clinical findings.	Page 4 lines 9-12
Timeline	7	Historical and current information from this episode of care organized as a timeline	Pages 3 and 4
Diagnostic Assessment	8a	Diagnostic testing (such as PE, laboratory testing, imaging, surveys).	Page 4 lines 12-18
	8b	Diagnostic challenges (such as access to testing, financial, or cultural)	NA
	8c	Diagnosis (including other diagnoses considered)	Pages 4 and 5
	8d	Prognosis (such as staging in oncology) where applicable	NA
Therapeutic Intervention	9a	Types of therapeutic intervention (such as pharmacologic, surgical, preventive, self-care)	Page 4 paragraph 2
	9b	Administration of therapeutic intervention (such as dosage, strength, duration)	Page 4 paragraphs 1 and 2
	9c	Changes in therapeutic intervention (with rationale)	Page 4 paragraph 2
Follow-up and Outcomes	10a	Clinician and patient-assessed outcomes (if available)	Page 4 lines 23 to 34
	10b	Important follow-up diagnostic and other test results	Page 4 lines 31 -34
	10c	Intervention adherence and tolerability (How was this assessed?)	NA
	10d	Adverse and unanticipated events	Page 4 lines 24 to 27
Discussion	11a	A scientific discussion of the strengths AND limitations associated with this case report	Page 6 lines 13 to 17
	11b	Discussion of the relevant medical literature **with references**.	Pages 5 and 6
	11c	The scientific rationale for any conclusions (including assessment of possible causes)	Pages 5 and 6
	11d	The primary "take-away" lessons of this case report (without references) in a one paragraph conclusion	Page 6 paragraph 2
Patient Perspective	12	The patient should share their perspective in one to two paragraphs on the treatment(s) they received	NA
Informed Consent	13	Did the patient give informed consent? Please provide if requested	Yes ☑ No ☐
